# Life on the Rocks: First Insights Into the Microbiota of the Threatened Aquatic Rheophyte *Hanseniella heterophylla*

**DOI:** 10.3389/fpls.2021.634960

**Published:** 2021-06-14

**Authors:** Witoon Purahong, Shakhawat Hossen, Ali Nawaz, Dolaya Sadubsarn, Benjawan Tanunchai, Sven Dommert, Matthias Noll, La-aw Ampornpan, Petcharat Werukamkul, Tesfaye Wubet

**Affiliations:** ^1^Department of Soil Ecology, UFZ-Helmholtz Centre for Environmental Research, Halle, Germany; ^2^Institute of Ecology and Evolution, Friedrich-Schiller-Universität Jena, Jena, Germany; ^3^Department of Community Ecology, UFZ-Helmholtz Centre for Environmental Research, Halle, Germany; ^4^Department of Civil, Geo and Environmental Engineering, Technical University of Munich, Garching, Germany; ^5^Institute for Bioanalysis, Coburg University of Applied Sciences and Arts, Coburg, Germany; ^6^Department of Biology, Srinakharinwirot University, Bangkok, Thailand; ^7^Faculty of Science and Technology, Rajamangala University of Technology Phra Nakhon, Bangkok, Thailand; ^8^German Centre for Integrative Biodiversity Research, Halle-Jena-Leipzig, Leipzig, Germany

**Keywords:** aquatic plant, microbiome, indicator species, anthropogenic disturbance, rocks, remediation

## Abstract

Little is known about microbial communities of aquatic plants despite their crucial ecosystem function in aquatic ecosystems. Here, we analyzed the microbiota of an aquatic rheophyte, *Hanseniella heterophylla*, growing at three areas differing in their degree of anthropogenic disturbance in Thailand employing a metabarcoding approach. Our results show that diverse taxonomic and functional groups of microbes colonize *H. heterophylla*. Proteobacteria, Actinobacteria, Dothideomycetes, and Sordariomycetes form the backbone of the microbiota. Surprisingly, the beneficial microbes reported from plant microbiomes in terrestrial habitats, such as N-fixing bacteria and ectomycorrhizal fungi, were also frequently detected. We showed that biofilms for attachment of *H. heterophylla* plants to rocks may associate with diverse cyanobacteria (distributed in eight families, including Chroococcidiopsaceae, Coleofasciculaceae, Leptolyngbyaceae, Microcystaceae, Nostocaceae, Phormidiaceae, Synechococcaceae, and Xenococcaceae) and other rock biofilm-forming bacteria (mainly *Acinetobacter*, *Pseudomonas*, and *Flavobacterium*). We found distinct community compositions of both bacteria and fungi at high and low anthropogenic disturbance levels regardless of the study areas. In the highly disturbed area, we found strong enrichment of Gammaproteobacteria and Tremellomycetes coupled with significant decline of total bacterial OTU richness. Bacteria involved with sulfamethoxazole (antibiotic) degradation and human pathogenic fungi (*Candida*, *Cryptococcus*, *Trichosporon*, and *Rhodotorula*) were exclusively detected as indicator microorganisms in *H. heterophylla* microbiota growing in a highly disturbed area, which can pose a major threat to human health. We conclude that aquatic plant microbiota are sensitive to anthropogenic disturbance. Our results also unravel the potential use of this plant as biological indicators in remediation or treatment of such disturbed ecosystems.

## Introduction

Aquatic plants perform many ecosystem functions in aquatic habitats and provide services to human society (García-Llorente et al., 2011). One of the important functions performed by aquatic plants is the uptake of dissolved nutrients (N and P) from water (Brix, 1997). They are widely used in constructed wetlands around the world to remove excess N and P from polluted water (Vymazal, 2013). Beside direct nutrient uptake, aquatic plants indirectly influence nutrient cycling, especially N cycling through influencing the denitrifying bacteria inhabiting in their roots and shoots (Hallin et al., 2015). Furthermore, aquatic plants promote the sedimentation of suspended solids by reducing the current velocities and impede erosion by stabilizing soil surfaces (Horppila et al., 2013; Zhu et al., 2015). Habitat complexity provided by aquatic plants is likely to increase the richness of taxonomy and density of both fish and invertebrates (Thomaz et al., 2008). Roots of aquatic plants provide extended surface for benthic microbial communities to rest and act as a customized niche for a diverse group of microbes ensuring the continuous supply of nutrients, organic carbon, and oxygen (Stottmeister et al., 2003).

Like terrestrial plants, aquatic plants living in aquatic environments also closely interact with microbial communities associated with their roots. In nature, healthy and asymptomatic plants cohabit with diverse and complex microbial consortia (Berendsen et al., 2012; Vorholt, 2012), which actively contribute to plant growth promotion, nitrification, denitrification, and remediation of contaminants (Ottosen et al., 1999; Yamaga et al., 2010; Tanaka et al., 2012). Although plants have adapted to alleviate most biotic and abiotic stresses in nature, they also rely on their microbial partners to survive and defend themselves against microbial invaders (Turner et al., 2013). Microbial assemblages (especially bacteria) are found as biofilms on solid substrata and plant surfaces especially on aquatic plants (Gagnon et al., 2007; Srivastava et al., 2017). Bacterial biofilms are important for adaptation, nutrient uptake, and survival of aquatic plants (Morris and Monier, 2003). Excessive nutrients (eutrophication) (Giaramida et al., 2013) and the presence of toxic substances in the water due to anthropogenic activities significantly affect biofilms and their structures (Calheiros et al., 2009; Srivastava et al., 2017). Hence, this may strongly affect the plant–microbe interactions and subsequently impacts aquatic plant growth and survival.

Rheophytes are aquatic plant species confined to the beds of fast-running streams and rivers, tightly attached to streambed substrates, especially rocks (Imaichi and Kato, 1997). Due to their special habitat, they have been poorly studied as compared with terrestrial plants and other aquatic plants (Werukamkul et al., 2016). This limits knowledge pertaining to their flora and associated microbial communities. *Hanseniella heterophylla* is a rear rheophilic herb from the family Podostemaceae and is considered a threatened plant species (vulnerable, VU) according to the International Union for Conservation of Nature (IUCN). *H*. *heterophylla* is endemic to northern and northeastern Thailand. It occurs in a total of six rivers in Phitsanulok and Loei provinces (Kita and Kato, 2004; Werukamkul et al., 2016). The major population of *H*. *heterophylla* occurs in Khek river where the plants grow densely and rapidly on rocks. However, due to disturbances and pollution, the population decline is estimated to be 20% at Kaeng Sopha Waterfall located in Khek river (Kita and Kato, 2004). As a rheophyte growing on the rocks, *H*. *heterophylla* has many challenges for their survival including nutrient limitation, extreme water currents, water availability, and seasonal changes. *H*. *heterophylla* is one of the rheophyte species in which the developing root became lobed and produced additional tufts of leaves in its more distal part. The leaf is filiform with thin dense hairs on the ventral side; by this mechanism, they adapt themselves to the rock surface and extreme water current (Koi et al., 2012). Similar to other plant species in Podostemaceae, *H*. *heterophylla* develop so-called adhesive hairs on its undersurface. These adhesive hairs have never been found to be in direct contact with the surface of rocks, but they are imbedded in biofilms (thickness = 100 μm or more) of diverse bacteria and cyanobacteria occurring on rocks (Morris and Monier, 2003). These biofilms serve as pasting glue for attachment of the plants to rocks that allows the plants to cope with an enormous tensile stress of the fast-running water in streams and rivers (Jäger-Zürn and Grubert, 2000). Bacterial and cyanobacterial biofilms are thus considered vital for survival of *H*. *heterophylla* as well as other Podostemaceae plants (Jäger-Zürn and Grubert, 2000; Morris and Monier, 2003). However, the information on the taxonomy of bacteria and cyanobacteria associated with *H*. *heterophylla* is still unknown.

Despite the potential importance of microbes for rheophyte existence, surprisingly there is no study on the microbiota (including bacteria and fungi). This highlights the need to explore the microbiota of *H*. *heterophylla* and to investigate the interplay of growing locations and local environment/anthropogenic disturbance on their microbiota. Therefore, in the present study, we analyzed the microbiota of the threatened aquatic rheophyte (*H*. *heterophylla*) using paired-end Illumina sequencing of the bacterial 16S and the fungal nuclear ribosomal internal transcribed spacer (ITS2) region. Our study aimed to (i) characterize the microbiota of *H*. *heterophylla*, (ii) investigate the rock bacterial community that may contribute to the biofilm formation for attachment of the plants to rocks that allows them to cope with the fast-running water environment, (iii) investigate the microbes that may potentially help *H*. *heterophylla* to overcome the nutrient limitation in aquatic environment, (iv) investigate the effects of location and local environment (sampling location, disturbance level, and rock types) on the *H*. *heterophylla* microbiota, and (v) identify the microbial indicators to the disturbance and sampling locations. We hypothesized that rock types, locations, and disturbance level impact the microbiota of *H*. *heterophylla*. We also hypothesized that bacterial genera (i.e., *Acinetobacter*, *Aeromonas*, *Arthrobacter*, *Bacillus*, *Chryseobacterium*, *Geodermatophilus, Flavobacterium*, *Paracoccus*, *Pseudomonas*, *Rhizobium*, *Serratia*, *Solibacillus*, and *Yersinia*) as well as cyanobacteria that are known to form biofilms on the surface of rocks are frequently detected in the microbial communities associated with *H*. *heterophylla* (Morris and Monier, 2003; Gorbushina, 2007; Chiellini et al., 2019). Concerning the nutrition of the plant, we hypothesize that, like terrestrial plants, some bacteria and fungi such as N-fixing bacteria and mycorrhizae may live symbiotically with *H*. *heterophylla*. We expect to detect *nifH* gene in DNA extracted from *H*. *heterophylla*.

## Materials and Methods

### Study Areas and Sampling Design

*Hanseniella heterophylla* were collected from rocks in five out of six rivers (a total of three study areas) in northern (Phitsanulok province, two study areas) and northeastern (Loei province, one study area) Thailand, where it naturally grows. In general, *H*. *heterophylla* is growing on both sandstone and siltstone. The field sampling was conducted in April 2015. At each study area, five sampling points with minimum distance of 300–500 m were selected. The two study areas in Phitsanulok province (Supplementary Figure 1) were established as (i) **Khek river** (later on refers to area 1: Khek river) and (ii) **Than**, **Ton**, and **Namkan rivers** (later on refers to area 2: Than river). These three rivers merge into Khek river (first study area). One study area in Loei province (Supplementary Figure 2) was established along **San river** (later on refers to area 3: San river). The first study area (Khek river) was classified as the highest anthropogenic disturbed area with whitewater rafting activities, wastewater from local restaurants, and chemical pollutions from agriculture while the other two sampling areas are located at the inner part of forests and/or rural area with low population with much less anthropogenic disturbance. In this study, five types of sedimentary and metamorphic rocks are present in the sampling area including jpk, purple or purple-red siltstone with gray-green and yellow-brown sandstone; kkk, red and brown-red siltstone and sandstone; ksk, brown-red, purple, and red siltstone and sandstone with high calcrete; ktky, red to brown-red sandstone; ktpk, mixture of brown-red sandstone, siltstone, mudstone, and conglomerate. The types of sedimentary and metamorphic rocks at the sampling area were identified according to the Department of Mineral Resources, Thailand. Descriptions (locations, co-ordinates, rock types, and disturbance levels) regarding the three study areas are shown in Table 1.

**TABLE 1:** Characteristics and anthropogenic disturbance levels of sampling locations, sampling areas, and sampling points.

ID	Location	Study area	Sampling point	Characteristics	Rock type*
S1 S2 S3 S4 S5	Phitsanulok province, Northern Thailand (16°52′49.7″1 N, 100°50′05.7″ E)	Khek river Khek river Khek river Khek river Khek river	Kaeng Sopha Waterfall Kaeng Pakhao Krayang Kaeng Yao Kaeng Ratchamung Kaeng Tinthai Bon	Highest anthropogenic disturbance level: located near the city with the presence of whitewater rafting activities, wastewater, and agricultural chemical pollutions	kkk ksk jpk jpk jpk
S6 S7 S8 S9 S10	Phitsanulok province, Northern Thailand (17°14′51.7″ N, 100°53′38.1″E)	Than river Than river Than river Than river Than river	Wanglum Waterfall (Ton river) Tadtam Waterfall (Ton river) Tadtinmee Waterfall (Ton river) Tintok Waterfall (Than river) Kaeng Huataek (Namkan rivers)	Low anthropogenic disturbance level: located at the inner part of forests	ktpk ktpk ktpk ktky kkk
S11 S12 S13 S14 S15	Loei province, Northeastern Thailand (17°28′03.7″ N, 101°16′48.2″ E)	San river San river San river San river San river	Kaeng Gliang 1 Kaeng Gliang 2 Kaeng Gliang 3 Kaeng Pak Nao 1 Kaeng Pak Nao 2	Low anthropogenic disturbance level: located at the inner part of forests/rural area with low population	kkk kkk kkk kkk kkk

*Rock types: jpk, purple or purple-red siltstone with gray-green and yellow-brown sandstone; kkk, red and brown-red siltstone and sandstone; ksk, brown-red, purple, and red siltstone and sandstone with high calcrete; ktky, red to brown-red sandstone; ktpk, mixture of brown-red sandstone, siltstone, mudstone, and conglomerate.

### Sampling, Sample Processing, and DNA Extraction

At each sampling point, we randomly assigned three subplots (30 cm × 30 cm) on rocks where *H*. *heterophylla* was growing. *H*. *heterophylla* plants were collected from all corners and at the middle of all subplots with knife and combined as one composite sample per sampling point. *H*. *heterophylla* samples were cleaned, stripped of all debris, and shipped on ice to a molecular laboratory. *H*. *heterophylla* samples (including all parts of the plants) were intensively washed using Milli-Q water (three times, 2 min vortex), 70% ethanol (one time, 3 min), Milli-Q water (three times), and PCR water (one time, 1 h and vortex 2 min). Each *H*. *heterophylla* sample was homogenized and genomic DNA from each sample was extracted from 150 mg of the homogenized sample using the ZR Soil Microbe DNA MiniPrep kit (Zymo Research, Irvine, CA, United States) following the manufacturer’s instructions. DNA quality and quantity were measured by spectrophotometric quantification with a NanoDrop ND-8000 V1.1.1 spectrophotometer (Thermo Fisher Scientific, Dreieich, Germany). DNA extracts were then stored at −20°C for further analysis.

### Microbial Community Analysis

Bacterial and fungal amplicon libraries were obtained separately for Illumina sequencing using the primer combination 799F (5′AACMGGATTAGATACCCKG3′) (chloroplast excluding) and 1115R (5′AGGGTTGCGCTCGTTRC3′), which target the V5–V6 region of the bacterial 16S rRNA gene (Chelius and Triplett, 2001; Redford et al., 2010; Beckers et al., 2016), and fITS7 (5′GTGARTCATCGAATCTTTG3′) (Ihrmark et al., 2012), and ITS4 (5′TCCTCCGCTTATTGATATGC3′) (White et al., 1990), which target the fungal ITS2 region. The PCR reaction mix included 1–10 ng of DNA extract as template (total volume 1 μl) and 15 pmol of each forward primer (799F for bacteria and fITS7 for fungi) and reverse primer (1115R for bacteria and ITS4 for fungi) in 20 μl volume of MyTaq buffer containing 1.5 units of MyTaq DNA polymerase (Bioline) and 2 μl of BioStabII PCR Enhancer (Sigma, United States). For each sample, the forward and reverse primers had the same 10-nt barcode sequence. PCRs were carried out for 30 (bacteria) or 35 (fungi) cycles using the following parameters: 1 min 96°C pre-denaturation; 96°C for 15 s, 55°C for 30 s, and 70°C for 90 s. The concentration of amplicons was assessed by gel electrophoresis. For bacterial amplicon libraries, a ∼445-bp fragment was isolated and extracted from agarose gel. About 20-ng amplicon DNA of each sample was pooled for up to 48 samples carrying different barcodes. The amplicon pools were purified with one volume Agencourt AMPure XP beads (Beckman Coulter, Inc., IN, United States) to remove primer dimer and other small mispriming products, followed by an additional purification on MiniElute columns (QIAGEN GmbH, Hilden, Germany). About 100 ng of each purified amplicon pool DNA was used to construct Illumina libraries using the Ovation Rapid DR Multiplex System 1-96 (NuGEN Technologies, Inc., CA, United States). Illumina libraries (Illumina, Inc., CA, United States) were pooled and size selected by preparative gel electrophoresis. Sequencing was done on an Illumina MiSeq using V3 Chemistry at LGC Genomics Berlin, Germany.

### Bioinformatics

The raw reads were first quality filtered for high-quality reads from the paired-end sequences generated by the Illumina MiSeq sequencing platform using MOTHUR (Schloss et al., 2009) and OBI Tools (Boyer et al., 2016) software suites. Forward and reverse raw reads from the same sample were assembled by using the simple Bayesian algorithm with a threshold of 0.6 and a minimum overlap of 15 (fungi) or 20 (bacteria) nucleotides as implemented in PANDAseq (Masella et al., 2012). Reads fulfilling the following criteria were retained for further analyses: a minimum length of 250 (bacteria) and 200 (fungi) nt, a minimum average quality of 26 (bacteria) or 25 (fungi) Phred score, containing homopolymers with a maximum length of 10 nt, and without ambiguous nucleotides. We then pre-clustered the reads using CD-HIT-EST, using a maximum of 1% of dissimilarity and with only one base allowed per indel (Huang et al., 2010), in order to merge those reads arising likely from sequencing errors (Huse et al., 2014). We detected chimeric sequences using the UCHIME algorithm (Edgar et al., 2011) as implemented in MOTHUR and removed from the datasets. The obtained reads were then clustered into operational taxonomic units (OTUs) using the CD-HIT-EST algorithm (Fu et al., 2012) at a threshold of 97% sequence similarity. The OTU representative sequences (defined as the most abundant sequence in each OTU) were taxonomically assigned using the SILVA database (v 128 and 138) for prokaryote 16S (Quast et al., 2013) and the UNITE database (v 7.0) (Kõljalg et al., 2013) for fungi, using the naive Bayesian classifier (Wang et al., 2007) as implemented in MOTHUR using the default parameters. Additionally, all the sequences identified as fungi were again classified against fungal sequences of the UNITE database augmented with non-fungal eukaryotic sequences from National Center for Biotechnology Information (NCBI) (version 211) (Benson et al., 2013) in order to detect sequences from non-target organisms. Rare OTUs (singleton to tripletons in the global matrix) that potentially might originate from artificial sequences (Kunin et al., 2010) were removed. The read counts were normalized to the smallest read number per sample (4325 reads for bacteria and 5519 reads for fungi). The final normalized dataset without rare OTUs was used for further statistical analysis unless otherwise stated. Numbers of 16S and ITS sequence reads at different steps of bioinformatics workflow are shown in Supplementary Tables 1, 2. We used a Mantel test based on the Bray–Curtis distance measure with 5000 permutations to assess the correlations between the whole matrix and a matrix excluding the rare OTUs for both bacterial and fungal datasets (Purahong et al., 2016). The results indicated that the removal of rare OTUs from the bacterial and fungal communities had no effect (bacterial dataset: *R*_*Mantel*_ = 0.95, *P* = 0.001; fungal dataset: *R*_*Mantel*_ = 1.000, *P* = 0.001). Ecological functions were predicted for detected bacterial OTUs using FAPROTAX (Louca et al., 2016; Sansupa et al., 2021) and the functional Annotation tool of Prokaryotic Taxa and FUNGuild (Nguyen et al., 2016) for fungi. In total, we successfully assigned the functions to 382 bacterial (37%) and 287 fungal (34%) OTUs. The bacterial 16S and fungal ITS2 raw reads are deposited in the NCBI Sequence Read Archive (SRA) under bioproject number PRJNA681338^1^.

### Quantitative Real-Time PCR

The presence of nitrogen-fixing bacteria and the potential nitrogen-fixing activity in samples were accessed by quantitative real-time PCR (qPCR). The qPCR analysis was performed to determine the *nifH* gene copy number. Real-time PCR was conducted in C1000^TM^ Thermal Cycler and CFX96^TM^ Real-Time PCR detection Systems (Bio-Rad, Singapore). Genomic DNA extracted from a culture of *Azotobacter vinelandii* (DSM 2289) was used to establish quantification standards for generating a standard curve. DNA concentration (gene copies μl^–1^) was determined by Petroff counting chamber (Paul Marienfeld GmbH, Germany). A five-point 10-fold serial dilution of the *A. vinelandii* genomic DNA (10–100,000 fg) was run in triplicate with each set of reactions to generate the standard curve. The *nifH* primers (PolF/PolR) were used for qPCR according to Poly et al. (2001). The reactions were performed in 10-μl assays containing 5 μl of SYBR^®^ Green Supermix (Bio-Rad, Germany), forward and reverse primers [0.5 μl for each primer (2.5 μM)], 3 μl of sterile and nuclease-free water (Carl Roth GmbH, Germany), and 1 μl of either 1:10 diluted DNA extract or 10-fold diluted standard DNA. After an initial denaturation at 94°C for 5 min, 34 amplification cycles were performed for 1 min at 95°C (denaturation), 1 min at 55°C (annealing), and 1 min 30 s at 72°C (extension), followed by a final extension of 5 min at 72°C. To check for product specificity and potential primer dimer formation, runs were completed with a melting analysis starting from 65 to 95°C with temperature increments of 0.5°C and a transition rate of 5 s. The purity of the amplified products was checked by electrophoresis on a 1.5% agarose gel. In addition, we used positive controls to evaluate the amplifiability/inhibition of the nucleic acid extracts by spiking a subset of each environmental nucleic acid extract (non-dilution, with 1:10 and 1:100 dilution) with 1 μl of genomic nucleic acid extract from *A*. *vinelandii* as positive control. As result, we observed no inhibition as the ct value shifted for each decimal dilution step in the same ct gap compared to the genomic nucleic acid extract from *A. vinelandii* without addition of environmental nucleic acid extract. On the other hand, we used the same collection tubes excluding *H. heterophylla* and we did the same cleaning procedure and DNA extraction method as explained in section “Sampling, Sample Processing, and DNA Extraction.” Thereafter, we used the resulting extracts for *nifH* gene-based qPCR as negative control. However, these extract samples could not be amplified.

### Statistical Analysis

Statistical analyses were performed using the R software (R Core Team, 2020), PAST program v. 2.17c (Hammer et al., 2001), and SPSS (IBM SPSS Statistics 24, New York, NY, United States). All the analyses were conducted based on three study areas. The observed richness and diversity of OTUs were calculated for each sampling point using PAST. Individual rarefaction curves are presented in Supplementary Figure 3. Diversity was calculated using Simpson index (1-dominance) which measure “evenness” of the community from 0 to 1 (0 = one taxon dominates the community completely and 1 = all taxa are equally present) (Hammer et al., 2001; Kim et al., 2017). Since removing the rare taxa (singletons, doubletons, and tripletons) can affect the calculation of the Simpson index (1-dominance), we also calculated this index using rarified datasets from all bacterial and fungal OTUs (including rare taxa from original dataset; 4571 reads for bacteria and 5694 reads for fungi). In this study, we detected consistent results of bacterial and fungal diversity either when including or when excluding the rare taxa (Supplementary Figure 4). Non-metric multidimensional scaling (NMDS) using the Jaccard dissimilarity measure and variance partitioning analysis were carried out to visualize and analyze the compositions of microbial communities in relation to study areas, disturbance levels, and rock types (Oksanen et al., 2016). Permutational multivariate analysis of variance (NPMANOVA) based on Jaccard distance was performed to test the community compositions of total bacteria and fungi, cyanobacteria, and ectomycorrhizal fungi (ECMf) at three study areas. As relative abundances obtained by metabarcoding approach could not be used quantitatively, we mostly used presence/absence data for multivariate statistics. One-way analysis of variance (ANOVA) incorporated with Tukey’s *post hoc* test was performed to test the impact of study areas and disturbances on the bacterial and fungal diversity indices (OTU richness and diversity) and copy numbers of the *nifH* gene, whereas non-parametric Kruskal–Wallis tests incorporated with Mann–Whitney *U* test was performed for OTU richness of cyanobacteria and ECMf. All richness, diversity, and *nifH* gene copy number datasets were tested for normality and the equality of group variances using Jarque–Bera test and the Levene statistics. The best predictors for bacterial and fungal OTU richness were analyzed using multiple regression in SPSS based on stepwise selection. Heat maps of all OTUs assigned as fungi with function detected in *H. heterophylla* (with minimum threshold of 40%, presence/absence data) were created by the package pheatmap (Kolde and Kolde, 2015) in R software. Indicator species analysis for three study areas (with low and high levels of disturbance) was performed using the multipatt function of indicspecies package (De Cáceres, 2013) in R. Bonferroni-corrected *P*-values were applied for the indicator species analysis.

## Results

### Taxonomic and Functional Groups of the *H. heterophylla* Microbiota

**FIGURE 1:**
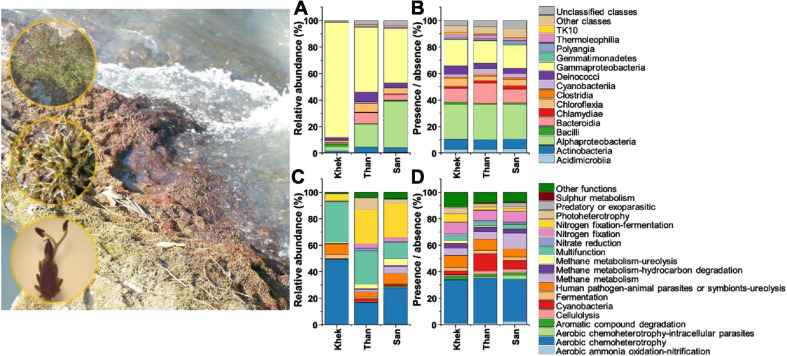
Taxonomic [class level, based on relative abundance **(A)** and presence/absence **(B)** data] and functional [based on relative abundance **(C)** and presence/absence **(D)** data] information of bacteria associated with *Hanseniella heterophylla* under three study areas (Khek, Than, and San).

**FIGURE 2:**
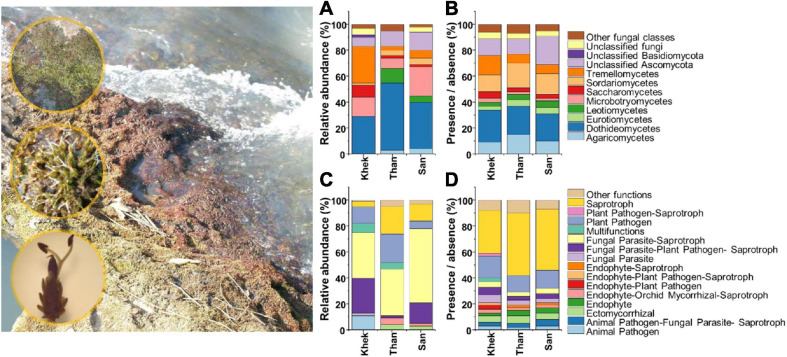
Taxonomic [class, based on relative abundance **(A)** and presence/absence **(B)** data] and functional [based on relative abundance **(C)** and presence/absence **(D)** data] information of fungi associated with *Hanseniella heterophylla* under three study areas (Khek, Than, and San).

A total of 64,875 quality-filtered bacterial 16S and 82,785 fungal ITS2 reads were obtained after removal of chimeric, non-target, and rare OTU sequences and sequence normalization. The final datasets contained 1031 bacterial OTUs (area 1 Khek river: 412 OTUs, area 2 Than river: 743 OTUs, and area 3 San river: 750 OTUs) and 836 fungal OTUs (Khek river: 350 OTUs, Than river: 441 OTUs, and San river: 579 OTUs) (Supplementary Table 3). There were 26% of total bacterial OTUs detected in all study areas whereas 4, 19, and 18% were specific for Khek, Than, and San rivers, respectively. There were 18% of total fungal OTUs detected in all study areas whereas 10, 14, and 29% were specific for Khek, Than, and San rivers, respectively (Supplementary Table 3). The 1031 bacterial and 836 fungal OTUs were assigned to 18 and 15 ecological functions according to FAPROTAX and FUNGuild, respectively (Figures 1, 2 and Supplementary Table 4).

Patterns of bacterial taxonomic and functional community compositions at three study areas were different (Figure 1). Than and San rivers (low anthropogenic disturbance) shared some features that strongly differed from Khek river (high anthropogenic disturbance). Specifically, Gammaproteobacteria (86%) had the highest relative abundance and was the most dominant class in Khek, while in the other two study areas (Than and San), the most abundant classes were shared between Gammaproteobacteria (Than: 49%, San: 41%) and Alphaproteobacteria (Than: 17%, San: 35%). The bacterial OTU rich functions (functions contain the highest number of bacterial OTUs) in Than and San rivers were dominated by aerobic chemoheterotrophy (Than: 94 OTUs, 34% of total functional assigned OTUs; San: 84 OTUs, 32%), cyanobacteria (Than: 35 OTUs, 13%; San: 22 OTUs, 6%), and methane metabolism (Than: 23 OTUs, 6%; San: 36 OTUs, 12%), while in Khek river, they belonged to aerobic chemoheterotrophy (55 OTUs, 33%), human pathogens: animal parasites or symbionts: ureolysis (13 OTUs, 9%), and N fixation (13 OTUs, 9%). Cyanobacteria was very rare or completely absent from some sampling points in Khek river. The most abundant bacterial functional groups (functions contain the highest number of bacterial relative abundances) in Khek river corresponded to aerobic chemoheterotrophy (49%) and multifunction (31%) while three other functional groups [N fixation (Than: 29%, San: 29%), aerobic chemoheterotrophy (Than: 17%, San: 28%), and multifunction (Than: 25%, San: 12%)] were frequently detected in Than and San, respectively.

The analysis of the fungal taxonomic community composition was in line with bacteria whose features are shared by Than and San rivers but strongly differed from Khek river (Figure 2). However, the abundant fungal functional groups were similar across all three study areas. Relative abundance data showed that Dothideomycetes (29%) and Tremellomycetes (28%) were the most frequently detected classes in Khek river, while in Than and San, they belonged to Dothideomycetes (Than: 52%, San: 36%) and Microbotryomycetes (Than: 8%, San: 22%). The fungal OTU rich functions in all three areas were saprotroph (Khek: 39 OTUs, 33%; Than: 86 OTUs, 48%; San: 91 OTUs, 47%) and plant pathogen (Khek: 20 OTUs, 17%; Than: 24 OTUs, 13%; San: 27 OTUs, 14%). The most abundant fungal functional groups in all study areas were fungal parasites–saprotroph (Khek: 35%, Than: 36%, San: 57%), plant pathogen (Khek: 13%, Than: 22%, San: 6%), and fungal–plant pathogen–saprotroph (Khek: 27%, Than: 2%, San: 16%).

### Factors Shaping the Microbial Richness and Community Compositions Associated With *H. heterophylla*

**FIGURE 3:**
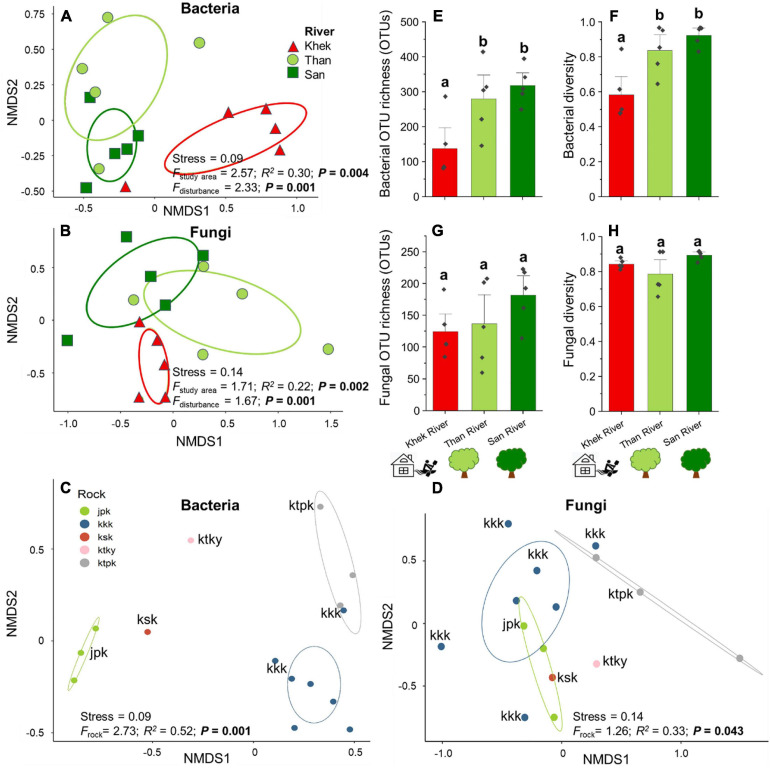
Effects of study areas, rock types, and anthropogenic disturbance levels on compositions and/or richness of microbial communities associated with *H*. *heterophylla*. Non-metric multidimensional scaling (NMDS) ordination diagrams of overall bacterial **(A)** and fungal **(B)** communities colonizing *H*. *heterophylla* in relation to study areas (Khek, Than, and San) and rock types—**(C)** bacteria and **(D)** fungi. NMDS based on Jaccard dissimilarities was used to determine the compositional variation; circles in the NMDS plot are 95% confidence ellipses. Effects of disturbance level on the richness and diversity (mean ± SE) of bacteria **(E,F)** and fungi **(G,H)** in *H*. *heterophylla.* Different letters above OTU richness bars within panels indicate significant differences (*P* < 0.05) according to one-way analysis of variance (ANOVA).

Bacterial OTU richness and diversity associated with *H. heterophylla* significantly declined (*F* = 6.44, *P* = 0.013 and *F* = 10.06, *P* = 0.003) in a highly disturbed area (Khek) as compared with the other two low disturbed areas whereas the fungal OTUs richness and diversity were not significantly different (*F* = 1.66, *P* = 0.230 and *F* = 1.04, *P* = 0.383) across three study areas (Figures 3E–H). Rock types also only significantly affected (*F* = 19.16, *P* < 0.001) bacterial richness and diversity (*F* = 29.01, *P* < 0.001) where jpk rock (purple or purple-red siltstone with gray-green and yellow-brown sandstone) had significantly lower OTU richness and diversity as compared with kkk (red and brown-red siltstone and sandstone) and ktpk (mixture of brown-red sandstone, siltstone, mudstone, and conglomerate) rocks (Supplementary Figure 5). Multiple regression analysis showed that disturbance level was the only significant predictor explaining large variations of bacterial OTU richness (adjusted *R*^2^ = 0.46, *F* = 12.85, *P* = 0.003) and diversity (adjusted *R*^2^ = 0.55, *F* = 6.71, *P* = 0.008). However, not any measured factor explained significant variations of fungal OTU richness. Community compositions of *H. heterophylla* microbiota (both bacteria and fungi) were affected by study areas (Bacteria: *F* = 2.57, *P* = 0.004 and Fungi: *F* = 1.71, *P* = 0.002) (Figures 3A,B) and rock types (Bacteria: *F* = 2.73, *P* = 0.001 and Fungi: *F* = 1.26, *P* = 0.043) (Figures 3C,D). Study areas and rock types explained large variations in bacterial (sampling areas = 30%, rock types = 52%) (Figures 3A,C) and fungal (sampling areas = 22%, rock types = 33%) (Figures 3B,D) community compositions. However, the NMDS analysis showed that the microbial community compositions (both bacteria and fungi) associated with *H. heterophylla* at Khek river were clearly distinct from the other two sampling areas. This result was confirmed by NPMANOVA, showing that the community compositions of microbiota associated with *H. heterophylla* in Khek river (highly disturbed area) was significantly different from Than and San rivers (Bacteria: *F* = 2.33, *P* = 0.001 and Fungi: *F* = 1.67, *P* = 0.001) (Figures 3A,B). Indeed, Khek and Than rivers are located closer together (within the same province and both rivers are connected) whereas San river is located >100 km away. Variation partitioning analysis confirmed that disturbance levels explained variations in bacterial and fungal communities that were not captured by the geographic locations/rock types (Table 2).

**TABLE 2:** Variation partitioning analysis to determine how disturbance levels, geographical locations, rock types, and their interactions explain variance in bacterial and fungal community compositions.

Factors	Bacteria		Fungi	
		
	Explained variance (%)	Percent of total explainable variance	Explained variance (%)	Percent of total explainable variance
Disturbance levels	1	7.1	1	12.5
Geographical locations	4	28.6	3	37.5
Rock types	5	35.7	0	0
Disturbance levels × geographical locations	1	7.1	0	0
Disturbance levels × rock types	2	14.3	1	12.5
Geographical locations × rock types	1	7.1	0	0
Disturbance levels × geographical locations × rock types	0	0	3	37.5

### Detections of Rock Biofilm-Forming Organisms

**FIGURE 4:**
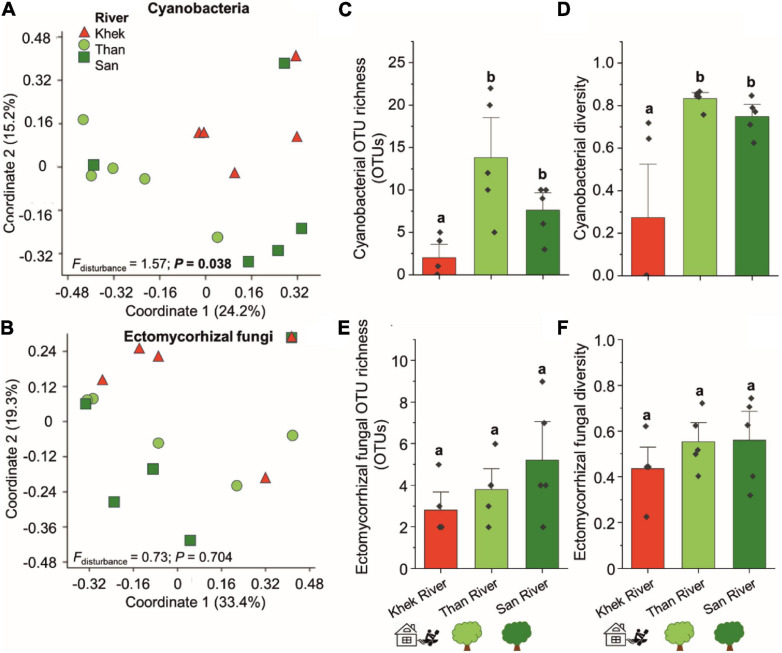
Effects of study areas and anthropogenic disturbance levels on compositions and richness of cyanobacteria and ectomycorrhizal fungi of *H*. *heterophylla*. Principal coordinates analysis (PCoA) of cyanobacteria **(A)** and ectomycorrhizal fungi **(B)** associated with *H*. *heterophylla* detected at different study areas including Khek (triangle), Than (circle), and San (square) rivers. PCoA based on Jaccard dissimilarities and presence/absence data was used to visualize the compositional variation. Effects of disturbance level on the richness and diversity (mean ± SE) of cyanobacteria **(C,D)** and ectomycorrhizal fungi **(E,F)** in *H*. *heterophylla.* Different letters above OTU richness bars within a panel indicate significant differences (*P* < 0.05) according to Kruskal–Wallis tests.

Bacterial genera known to form biofilms on the surface of rocks, including *Acinetobacter*, *Aeromonas*, *Arthrobacter*, *Chryseobacterium*, *Flavobacterium*, *Geodermatophilus*, *Paracoccus*, *Pseudomonas*, *Rhizobium*, and *Serratia*, were detected in the microbial communities associated with *H*. *heterophylla*. However, only *Acinetobacter*, *Pseudomonas*, and *Flavobacterium* were among the most frequently detected (all detected in more than 9 out of 15 sampling points) (Supplementary Table 4). *Pseudomonas* and *Acinetobacter* were ranked 2nd and 6th among the most detected bacteria in this study. *Pseudomonas* were highly detected in Khek river (high disturbance level) whereas *Flavobacterium* were mainly detected in low disturbance areas and absent from four out of five sampling points in Khek river. The analysis of the taxonomic composition of cyanobacteria in *H. heterophylla* revealed that a total of 38 cyanobacterial OTUs (all belonged to phylum Cyanobacteria) were detected in three study areas (Supplementary Table 5). They belonged to order Cyanobacteriales (19 OTUs, belonged to Chroococcidiopsaceae, Coleofasciculaceae, Microcystaceae, Nostocaceae, Phormidiaceae, and Xenococcaceae), Leptolyngbyales (nine OTUs, all belonged to Leptolyngbyaceae), Synechococcales (one OTU, Synechococcaceae), and unclassified cyanobacteria (eight OTUs) (Supplementary Table 5). We identified eight cyanobacterial genera, including *Tychonema*, *Pleurocapsa*, *Phormidesmis, Synechococcus*, *Calothrix*, *Chroococcidiopsis*, *Leptolyngbya*, and *Wilmottia*. *Synechococcus* and *Calothrix* were detected in all sampling areas. Surprisingly only eight OTUs were detected in Khek river as compared to Than (35 OTUs) and San (22 OTUs) rivers. By investigating the effect of the degree of disturbance, we found that the community composition, OTU richness, and diversity of cyanobacteria in Khek river (high disturbance) were significantly different from the other two study areas with low disturbance level (Figures 4A,C,D). The cyanobacterial OTU richness and diversity were significantly reduced (richness: *H* = 8.96, *P* = 0.0108, diversity: *F* = 9.18, *P* = 0.004) in a highly disturbed area (Khek river) as compared with lowly disturbed areas (Than and San) (Figures 4C,D). Also, NPMANOVA indicated that the cyanobacterial community composition was significantly different (*F* = 1.57, *P* = 0.038) between highly and lowly disturbed areas (Figure 4A). Additionally, we found two out of five sampling points in Khek river where the cyanobacteria were totally absent from the microbiota of *H. heterophylla*.

### Ectomycorrhizal Fungi—Richness and Taxonomic Information

From 836 fungal OTUs, we observed 11 OTUs assigned as ECMf (Figure 2D, Supplementary Table 6, and Supplementary Figure 6). Three OTUs including *Amanita*, *Russula fragilis*, and *Tomentella* were detected across three areas (Khek, Than, and San). Among them, *Tomentella* Otu0038 was the most frequently detected (15 out of 15 locations) followed by *Amanita* Otu0088 (9 out of 15 locations) and *R. fragilis* Otu0123 (8 out of 15 locations). Other taxa including *Chloridium, Lactarius* spp. (*Lactarius atromarginatus* and *Lactariu*s), and *Sebacinaceae* were only detected in Than and San rivers (lowly disturbed areas) and completely absent from Khek river. Richness, diversity, and community composition of ECMf were not significantly different (richness: *H* = 2.79, *P* = 0.227, diversity: *F* = 1.04, *P* = 0.383, and community composition: *F* = 0.73, *P* = 0.704) across different study areas (Figures 4B,E,F).

### Microbes That May Potentially Help Aquatic Plants to Uptake Nutrients

**FIGURE 5:**
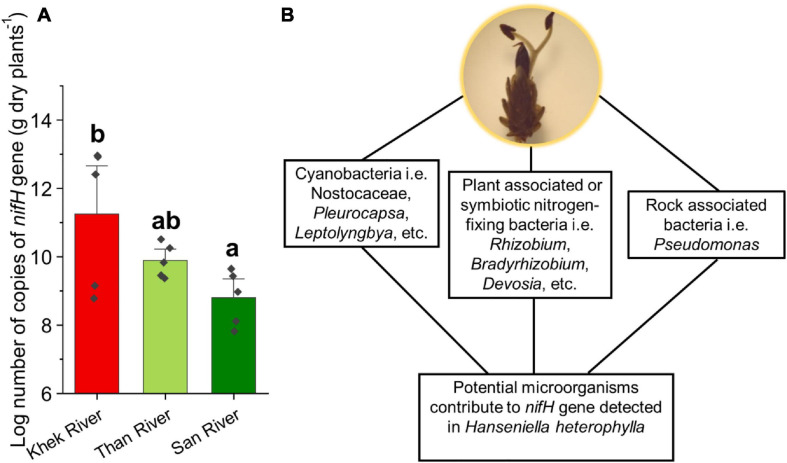
Copy numbers of the *nifH* gene detected in *H*. *heterophylla* across three study areas (Khek, Than, and San) **(A)** and potential microorganisms contribute to the presence of the *nifH* gene **(B)**. Different letters indicate significant differences (*P* < 0.05) according to one-way analysis of variance (ANOVA).

We frequently detected beneficial microbes reported from terrestrial plant microbiomes such as N-fixing bacteria, mycorrhizal, and endophytic fungi (Supplementary Figure 6 and Supplementary Table 4). Apart from the cyanobacteria, we found N-fixing bacteria belonging to *Bacillus* spp., *Devosia* spp., *Pantoea* spp., *Mycobacterium* spp., *Clostridium* spp., *Rhizobium* spp. (including *Rhizobium radiobacter*), *Bradyrhizobium elkanii*, *Azorhizobium* sp., *Mesorhizobium* sp., *Shinella* sp., *Microvirga* spp., *Azospirillum* spp., *Burkholderia-Paraburkholderia* sp., and *Derxia* sp. We further analyzed our samples to detect the *nifH* gene involved in fixation of the atmospheric nitrogen into a form available to living organisms by using real-time PCR. We detected high copy numbers of *nifH* gene in all the samples ranging from 6.55 × 10^7^ to 9.19 × 10^12^ copies per gram dry plant (Figure 5A). *H. heterophylla* in Khek river had significantly higher *nifH* gene copy numbers than San river (*F* = 4.28, *P* = 0.039). We also detected beneficial fungi, i.e., ECMf (see previous section), ericoid mycorrhizal (e.g., *Oidiodendron chlamydosporium, Pseudeurotium hygrophilum*), and plant growth-promoting endophytes [*Piriformospora indica, Trichoderma atroviride*, *Hypocrea lixii* (*Trichoderma harzianum*), etc.].

### Plant Pathogens Associated With *H*. *heterophylla*

In all three study areas (Khek, Than, and San), we detected potential fungal plant pathogens associated with *H. heterophylla*, i.e., *Mycosphaerella tassiana, Pseudocercospora* sp., *Gibberella fujikuroi, Curvularia lunata, Lasiodiplodia crassispora*, and *Clonostachys rosea* (Supplementary Table 6). Among them, *M. tassiana* was the most frequently detected plant pathogen. We also found some pathogens specific for Khek (highly disturbed area) including *Trichothecium* sp. and *Gibberella intricans.* On the other hand, *Plectosphaerella alismatis, Pestalotiopsis rhododendri, Edenia* sp., *Ceratorhiza hydrophila, Stagonospora* sp., *Mycosphaerella* sp., *Eutypella* sp., *Neodeightonia* sp., and *Pilidiella tibouchinae* were detected only in the lowly disturbed areas (Than and San rivers).

### Microbial Indicators of *H*. *heterophylla* Growing Under Different Disturbance Levels

We performed indicator species analysis and identified 2 bacterial OTUs and 9 fungal OTUs as indicator species in Khek river (high disturbance area), whereas 4 and 8 bacterial OTUs were found as indicators in Than and San rivers (low disturbance area), respectively (Table 3). Interestingly, there were no fungal indicators in the low disturbance areas. *Pseudomonas psychrophila*, bacterium involved with sulfamethoxazole (antibiotic) degradation and waste water as well as many human pathogenic fungi (*Candida zeylanoides, Trichosporon gracile, Rhodotorula minuta*, and *Candida parapsilosis*) were exclusively detected as indicator microorganisms in *H. heterophylla* growing in the highly disturbed area (Khek river). These fungal indicators are all identified as yeast. The list of indicators in the three study areas is shown in Table 3.

**TABLE 3:** Potential indicators found in three areas (Khek, Than, and San rivers) of *Hanseniella heterophylla*.

Study area	Microbial OTU	Function	Stat	*P*-value	References
Khek	*Pseudomonas psychrophila* Otu0001 (bacteria)	Aerobic chemoheterotrophy, sulfamethoxazole (antibiotic) degradation	0.99	<0.05	Jiang et al., 2014
	*Meiothermus* Otu0083 (bacteria)	Unassigned	0.89	<0.05	
	*Candida zeylanoides* Otu0009 (fungi)	Opportunistic yeast (human pathogen)	0.95	<0.05	Levenson et al., 1991
	*Cryptococcus victoriae* Otu0012 (fungi)	Associated with mosses, lichens, and soils	0.88	<0.05	Montes et al., 1999
	*Trichosporon gracile* Otu0018 (fungi)	Human pathogen	0.89	<0.05	Mariné et al., 2015
	Basidiomycota Otu0026 (fungi)	Unidentified	0.89	<0.05	
	Fungi unclassified Otu0029 (fungi)	Unidentified	0.99	<0.05	
	*Cryptococcus cyanovorans* Otu0041 (fungi)	Associated with cyanide-contaminated soil	1	<0.01	Motaung et al., 2012
	*Cystobasidium slooffiae* Otu0075 (fungi)	Fungal parasite	0.99	<0.01	Kurtzman et al., 2011
	*Rhodotorula minuta* Otu0081 (fungi)	Human pathogen	1	<0.01	Wirth and Goldani, 2012
	*Candida parapsilosis* Otu0114 (fungi)	Human pathogen	0	<0.01	Trofa et al., 2008
Than	*Cloacibacterium* Otu0019 (bacteria)	Unassigned	0.88	<0.05	
	*Fibrella* Otu0081 (bacteria)	Unassigned	0.89	<0.05	
	Pseudonocardiaceae Otu0089 (bacteria)	Unassigned	0.88	<0.05	
	*Roseomonas* Otu0096 (bacteria)	Human pathogen, animal parasites or symbionts, and ureolysis	0.96	<0.05	
San	Methylophilaceae Otu0060 (bacteria)	Methane metabolism, chemoheterotrophy	0.96	<0.01	
	Hyphomicrobiaceae Otu0088 (bacteria)	Unassigned	0.95	<0.01	
	*Hyphomicrobium* Otu0095 (bacteria)	Aerobic chemoheterotrophy	0.93	<0.05	
	*Exiguobacterium* Otu0097 (bacteria)	Unassigned	0.94	<0.05	
	Hyphomicrobiaceae Otu0099 (bacteria)	Unassigned	0.89	<0.05	
	Chitinophagaceae Otu0100 (bacteria)	Unassigned	0.92	<0.05	
	*Hyphomicrobium* Otu0132 (bacteria)	Aerobic chemoheterotrophy	0.85	<0.05	
	Micavibrionales Otu0180 (bacteria)	Unassigned	0.98	<0.01	

## Discussion

In this work, we assessed the *H*. *heterophylla*-associated bacterial and fungal richness and their relative abundances, and identified their functional groups across three study areas (Khek, Than, and San) and different levels of anthropogenic disturbance. Our analysis revealed that rock types, study areas, and levels of disturbance significantly impact the microbiota of *H*. *heterophylla*. We also identified the microbial indicators at low and high levels of anthropogenic disturbance. The microbial indicators of high disturbance levels were related to bacteria associated with wastewater and fungal (yeast) human pathogens (Table 3).

### Microbiota of *H. heterophylla* as Compared to Terrestrial Plant Models and Crops

Here, we used *H. heterophylla* as a model plant to investigate its associated microbiota and discuss literature comparing its microbiota to the other model plants and crops in terrestrial ecosystem including *Arabidopsis thaliana* (Schlaeppi et al., 2014; Bergelson et al., 2019), *Oryza* spp. (rice) (Edwards et al., 2015; Ding et al., 2019) and *Zea mays* (maize) (Peiffer et al., 2013), and the model forest tree genus *Populus* (Cregger et al., 2018). Overall, we found that the microbial communities of *H. heterophylla* had both unique and shared features with the other model plants in terrestrial ecosystems. In our study, the main bacterial classes detected in all growing areas of *H*. *heterophylla* are Alphaproteobacteria and Gammaproteobacteria that also have been highly detected in those of the abovementioned model plants (Weinert et al., 2011; Lundberg et al., 2012; Peiffer et al., 2013; Edwards et al., 2015; Pfeiffer et al., 2017; Cregger et al., 2018; Bergelson et al., 2019; Ding et al., 2019). Dothideomycetes and Sordariomycetes were highly detected fungal classes among all sampling areas of *H*. *heterophylla* that have been reported to be enriched in the rhizosphere of terrestrial plants including *A. thaliana*, *Oryza* spp., *Z. mays*, and *Populus* spp. (Albrectsen et al., 2010; Yuan et al., 2011; Szilagyi-Zecchin et al., 2016; Urbina et al., 2018). We also identified unique features as compared to other model plants. In other model plants, the relative abundance of Chloroflexi is significantly high, while in *H. heterophylla*, we found much lower relative abundance (Sui et al., 2019). Normally, in terrestrial model plants, Proteobacteria, Acidobacteria, and Actinobacteria form the backbone of the bacterial rhizosphere microbiota (Purahong et al., 2019). However, we rarely detected Acidobacteria (relative abundance <1%) and Actinobacteria (relative abundance ∼4%) in *H. heterophylla*. In general, the mycobiome of *H. heterophylla* (at class level) follows the common patterns of terrestrial model plants and crops. In contrast, the bacterial microbiota of *H. heterophylla* is more unique, with Alphaproteobacteria and Gammaproteobacteria forming the community backbone, along with a lower relative abundance of Acidobacteria, Actinobacteria, and Chloroflexi.

### Biofilms for Attachment of *H. heterophylla* Plants to Rocks May Associate With Diverse Cyanobacteria and Other Rock Biofilm-Forming Bacteria

It has been reported that cyanobacteria provide biofilms for attachment of Podostemonad plants to the rocks and can make a symbiotic relationship with plants or lichen-forming fungi (Dodds et al., 2008). Apart from cyanobacteria, some previous studies demonstrate that such rock biofilms are also composed of other bacterial groups (Gorbushina, 2007; Chiellini et al., 2019). Our current study shows that some rock biofilm-forming bacterial genera, including *Acinetobacter*, *Pseudomonas*, and *Flavobacterium*, are frequently detected or even dominant in *H*. *heterophylla* associated bacterial communities. *Acinetobacter* and *Pseudomonas* have been reported as the most common bacteria associated with black and red epilithic biofilm (epilithons) sampled from natural rock waterfall (Chiellini et al., 2019). In aquatic systems, microbial biofilms make a firm association around the aquatic plants, which facilitates the mutual supplies of nutrients (e.g., microbes interact with the plants for organic carbon and oxygen, whereas plants receive mineral exchange) (Srivastava et al., 2017). In this study, we detected diverse cyanobacteria distributed across eight families. Cyanobacteria not only may be important as biofilm producers for *H*. *heterophylla* to attach with rocks but also may provide additional N to the host plant. For instance, *Pleurocapsa* and *Synechococcus* are reported to be able to fix N (Waterbury and Stanier, 1978; Bergman et al., 1997). Importantly, we found an evidence that anthropogenic disturbance may endanger the association between cyanobacteria and *H. heterophylla.* In the highly disturbed area (Khek river), the richness and community composition of cyanobacteria are significantly reduced and changed. In some locations at Khek river, we did not detect any cyanobacteria. This situation may threaten *H*. *heterophylla* even more in highly disturbed areas. Nevertheless, in light of the primer pairs used to exclude chloroplast sequences and reports from other publications on reduction of cyanobacteria coverage using these primers (Chelius and Triplett, 2001; Redford et al., 2010; Beckers et al., 2016), reliably discussing cyanobacterial diversity and abundance is difficult here. Thus, though the question of whether “cyanobacteria are absent from Khek or that their interaction with *H. heterophylla* is somewhat impaired at that site” is very interesting, we decided to address this question in a future study.

### Detection of ECMf Associated With *H*. *heterophylla*

We detected common terrestrial ECMf associated with *H*. *heterophylla*. The function of ECMf in an aquatic habitat may much more differ compared to the terrestrial ecosystem. In the terrestrial ecosystem, ECMf extend their mycelium from the mantle into the surrounding soil to mineralize the soil organic matter and rock, which improves the availability of P and N to host plants (Agerer, 2006; Fernandez et al., 2016). In contrast, in the aquatic ecosystem with fast running water like in our study, we believe that ECMf may not extend their mycelium into surrounding water (to avoid damage) to get nutrients but rather go into the rocks. In the terrestrial ecosystem, ECMf can act as rock-eating fungi that mobilize nutrients from the rocks and probably form micropores through excretion of organic acids at their hyphal tips (Jongmans et al., 1997). We detected *Amanita* sp. and *Tomentella* sp. in our three study areas, which are reported as rock-eating fungi (Rosling, 2003; Sun et al., 2019). We propose that ECMf colonize and mineralize the rock and then transfer nutrients for *H*. *heterophylla*. *Amanita* sp., *Tomentella* sp., and *R. fragilis* are tolerant against the anthropogenic disturbance but other taxa including *Chloridium, Lactarius* spp., and *Sebacinaceae* are much more sensitive.

### Insights Into the Microbes That May Potentially Help Aquatic Plants to Uptake Nutrients

The depletion of phosphorus, and N, in aquatic plants appears to reflect a greater degree of P and N limitation that could lead to large changes in relative plant growth in an aquatic ecosystem (Duarte, 1992; Moe et al., 2019). We found beneficial microbiota including N-fixing bacteria, ECMf, and plant growth-promoting endophytic fungi in microbiota associated with *H*. *heterophylla*. We found very rich genera of N-fixing bacteria (14 genera) associated with *H*. *heterophylla* as compared with well-known legume plants (18 genera). Most N-fixing bacteria associated with *H. heterophylla* are similar to those reported in symbiotic N fixation of legume plants (MacLean et al., 2007; Velazquez et al., 2017). The exceptions are *Bacillus* spp., *Pantoea* spp., *Mycobacterium* spp*., Clostridium* spp., and *Azospirillum* spp. that are also fixing N in non-legume plants and/or in soil as asymbiotic N-fixing bacteria (Franche et al., 2009). Indeed, we detected high copy numbers of *nifH* gene in all sampling points across the three sampling areas. These numbers are similar to the values reported for soils collected in the vicinity of legumes (Barros et al., 2018). There are different groups of potential microorganisms that may contribute to the presence of *nifH* gene detected in *H. heterophylla*, including cyanobacteria, plant-associated or symbiotic N-fixing bacteria, and rock-associated bacteria (Figure 5B). We suggest that these bacterial groups may play an important role in N acquisition for *H*. *heterophylla*.

We detected the potential ECMf (*Amanita* spp. and *Tomentella* spp.) that may contribute to organic P mobilization from rocks to plant (Sawyer et al., 2003; Plassard et al., 2011). Apart from these, we also detected plant growth-promoting fungal endophytes [*P. indica, T. atroviride*, *H. lixii* (*T. harzianum*), etc.]. The endophytic fungus *P. indica* has dynamic functions (nutrient uptake, promotion of plant growth, protection, and stress tolerance) and exhibits its versatility in colonizing the plant species (Gill et al., 2016). *T. harzianum* and *T. atroviride* help plants to solubilize and take up nutrients as well as help in defense against root pathogens that has been reported in the study of many terrestrial plants (Ousley et al., 1993; Chaverri and Samuels, 2002; Macías-Rodríguez et al., 2018). Thus, these microbes could help *H. heterophylla* in nutrient acquisition.

### Rock Type Affects the Microbial Communities Associated With *H. heterophylla*

Microbiota of *H*. *heterophylla* potentially come from (i) endophytes, (ii) surrounding water, (iii) air, and/or (iv) rocks. The presence of plants on the rocky surface usually increases the nutrient-processing efficiency of aquatic habitats (Shilton, 2006). There is evidence that different rock types are associated with different microbial communities (Choe et al., 2018); thus, *H*. *heterophylla* plants growing on different rock types will be exposed to different pools of microorganisms. Rock filters rapidly acquire biofilm coatings on the rocky surface and these presumably act to entrap different microbial communities (Shilton, 2006). Various studies reported that rocks block the upward movement of water where aquatic plant roots and their associated microbiota act as drivers of mineral weathering, nutrient cycling, and ecosystem stability (Stark, 1970; Nobel et al., 1992; Burghelea et al., 2015). These would, therefore, underline our finding that rock types explained large variation in bacterial and fungal community composition associated with *H*. *heterophylla.*

### Level of Disturbance Impacts the *H*. *heterophylla* Microbiota

In this study, we demonstrated that high level of anthropogenic disturbance (e.g., agriculture and household wastewater and water sport activities at Khek river) significantly reduces richness and changes the community composition of microbes associated with *H*. *heterophylla* as compared to lowly disturbed areas (Than and San rivers). Furthermore, the anthropogenic level is the best predictor for bacterial OTU richness in this study. Consistent with the results of Santillan et al. (2019), disturbance level is reported to alter diversity as well as function of bacterial and fungal communities. Richness, relative abundance, and/or presence of specific microbial functional groups (especially the beneficial ones, i.e., cyanobacteria and ECMf) are also significantly reduced with increased disturbance level. These changes could affect the growth and adaptability of *H*. *heterophylla* to relatively low nutrient aquatic environments and the enormous tensile stress of the fast-running water (Roszak and Colwell, 1987; James et al., 1999).

### Microbial Indicators of the Level of Disturbance of *H*. *heterophylla* Growing Aquatic Systems

The microbial indicators of *H*. *heterophylla* microbiota revealed a pattern of bacterial and fungal taxa assigned as indicators of the level of disturbance. Focusing on the highly anthropogenic disturbed area (Khek river), we identified bacteria involved with antibiotic degradation as well as many human pathogenic fungi as microbial indicators. *P. psychrophila* (bacterial indicator) is associated with sulfamethoxazole degradation where bacteria uses sulfamethoxazole as the sole source of carbon and energy. Sulfamethoxazole is a common antibiotic that is frequently detected in wastewater and surface water as it is extensively used in both human and veterinary medicine (Jiang et al., 2014). Therefore, sulfamethoxazole is usually considered as an indicator of antibiotic pollution, and the presence of sulfamethoxazole in aquatic environments may pose long-term threats to aquatic and surrounding life (Jiang et al., 2014). In this work, we found this bacterial indicator in a highly disturbed area (Khek river), thereby demonstrating that this area may be polluted by manifold antibiotics including sulfamethoxazole. We also found fungal indicators, which are known as human pathogens including *C. zeylanoides* (*C. zeylanoides* fungemia) (Levenson et al., 1991), *T. gracile* (white piedra, hypersensitivity pneumonitis, superficial infections, and invasive trichosporonosis) (Mariné et al., 2015), *R. minuta* (meningeal, skin, ocular, peritoneal, and prosthetic joint infections) (Wirth and Goldani, 2012), and *C. parapsilosis* (invasive candidal disease) (Trofa et al., 2008). On the other hand, the microbial indicators (bacteria) of the lowly anthropogenic disturbed area were characterized by several functions including aerobic chemoheterotrophy and methane metabolism. The exception was *Roseomonas*, identified as potential human pathogen, animal parasites or symbionts, and ureolytic agent. As the identification is at the genus level, we cannot indicate the true function of this *Roseomonas* OTU. Our findings highlighted the potential role and use of *H*. *heterophylla* plant in bioremediation/phytoremediation in accordance with the established role of aquatic macrophytes to remove organic and inorganic pollutants from aquatic environments affected with various kinds of anthropogenic activities (Dhir et al., 2009).

## Conclusion

We reported the microbiota of *H*. *heterophylla* along different disturbance levels and found close interaction between *H*. *heterophylla* microbiota and the surrounding environmental factors. Our analyses showed a negative impact of anthropogenic disturbance on *H. heterophylla*-associated microbial communities, which could potentially be associated with the reduction of its population size. Furthermore, *H. heterophylla* growing in high anthropogenic disturbed areas can integrate many of the human pathogens into its microbiota, which poses a major threat to human health. This situation reinforces the need to monitor the richness and community compositions of the microbiota associated with diverse aquatic plants under different disturbance conditions. This will open the possibility to trap microbial water contaminants in plant roots in order to use those aquatic plants as bioremediation agents. However, this needs to be well studied and optimized to not compromise the growth and survival of the aquatic plant species with increasing disturbance and pollution.

## Sampling Permission

Sampling permission (permission number: 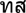 0904.4/22828) for collecting *Hanseniella heterophylla* was issued by the Department of National Park, Wildlife and Plant Conservation, Thailand, to PW.

## Data Availability Statement

The datasets presented in this study can be found in online repositories. The bacterial 16S and fungal ITS2 raw reads are deposited in the NCBI Sequence Read Archive (SRA) under bioproject number PRJNA681338: https://www.ncbi.nlm.nih.gov/bioproject/681338.

## Author Contributions

WP, PW, L-AA, and TW: conceptualization. WP and TW: methodology. PW and L-AA: field sampling and sample collection. WP, BT, SD, and DS: preparation of Illumina sequencing. AN and TW: bioinformatics. MN and SH: real-time PCR analysis. BT, SH, and WP: data analysis. TW: resources. WP and BT: data curation, visualization, and manuscript revision. SH and WP: writing—original draft preparation. SH, WP, AN, MN, and TW: writing—review and editing. WP: supervision. All authors have read and agreed to the published version of the manuscript.

## Conflict of Interest

The authors declare that the research was conducted in the absence of any commercial or financial relationships that could be construed as a potential conflict of interest.

## Footnotes

1 http://www.ncbi.nlm.nih.gov/bioproject/681338
