# Supplementary material for: Life on the Rocks: First Insights Into the Microbiota of the Threatened Aquatic Rheophyte Hanseniella heterophylla
Source: Front Plant Sci. 2021 Jun 14;12:634960. doi: 10.3389/fpls.2021.634960 (PMC8238419; doi:10.3389/fpls.2021.634960)

frontiers

Supplementary Material

Life on the rocks: first insights into the microbiota of the threatened aquatic rheophyte *Hanseniella heterophylla*

Witoon Purahong[1]†*, Shakhawat Hossen[1,2]*, Ali Nawaz[3,4]*, Dolaya Sadubsarn[1], Benjawan Tanunchai[1], Sven Dommert[1], Matthias Noll[5], La-aw Ampornpan[6], Petcharat Werukamkul[7]†*, Tesfaye Wubet[3,8]

Affiliations:

[1]UFZ-Helmholtz Centre for Environmental Research, Department of Soil Ecology, Halle (Saale), Germany

[2]Friedrich-Schiller-Universität Jena, Institute of Ecology and Evolution, Jena, Germany

[3]UFZ-Helmholtz Centre for Environmental Research, Department of Community Ecology, Halle (Saale), Germany

[4]Department of Civil, Geo and Environmental Engineering, Technical University of Munich, Garching, Germany.

[5]Institute for Bioanalysis, Coburg University of Applied Sciences and Arts, Coburg, Germany

[6]Srinakharinwirot University, Department of Biology, Bangkok, Thailand

[7]Rajamangala University of Technology Phra Nakhon, Faculty of Science and Technology, Bangkok, Thailand

[8]German Centre for Integrative Biodiversity Research (iDiv), Halle-Jena-Leipzig, Leipzig, Germany

***These authors contributed equally to this work.**

† Correspondence:

Corresponding Authors: witoon.purahong@ufz.de, petcharat.w@rmutp.ac.th

Table S1. Number of 16S sequence reads at different step of bioinformatics work flow.

Sample	Raw	Primer detected	Pair-end	Trimmed at 25 Phred score	All OTUs	Abundant OTUs	All Bacteria OTUs	Abundant Bacteria OTUs	Normalization	Mitochondria	Final rarified read
1	19354	9735	9506	7099	4573	4387	4571	4386	4386	0	4325
2	52491	25906	25262	19455	13431	13273	13429	13273	4386	1	4325
3	57438	28270	27722	22626	13919	13848	13919	13848	4386	3	4325
4	85400	42296	41482	33936	23711	23639	23711	23639	4386	0	4325
5	47647	23797	23304	18447	11432	11350	11430	11348	4386	3	4325
6	23840	11898	11621	8929	7458	7265	7454	7263	4386	28	4325
7	79139	39337	38447	30186	21131	20810	21128	20809	4386	0	4325
8	77472	39034	38131	30257	20433	19228	20304	19113	4386	21	4325
9	27051	13500	13178	9612	8341	8246	8340	8245	4386	4	4325
10	23111	11710	11366	8417	6298	6086	6291	6080	4386	1	4325
11	48647	23973	23408	18489	15527	15215	15520	15213	4386	4	4325
12	63457	31670	30965	24164	19037	18319	18970	18265	4386	2	4325
13	23561	11950	11557	8458	6187	6018	6183	6015	4386	8	4325
14	21585	10801	10521	8127	5795	5599	5786	5593	4386	47	4325
15	34946	17554	17131	13492	9052	8875	9045	8870	4386	61	4325
Minimum	19354	9735	9506	7099	4573	4387	4571	4386	4386	0	4325
Average	45675.9	22762.1	22240.1	17446.3	12421.7	12143.9	12405.4	12130.7	4386	12.2	4325
Maximum	85400	42296	41482	33936	23711	23639	23711	23639	4386	61	4325
Total	685139	341431	333601	261694	186325	182158	186081	181960	65790	183	64875

Table S2. Number of ITS sequence reads at different step of bioinformatics work flow.

Sample	Raw	Primer detected	Pair-end	Trimmed at 25 Phred score	All OTUs	Abundant OTUs	All Fungi OTUs	Abundant Fungi OTUs	Normalization	Mitochondria	Final rarified read
1	105187	52527	47144	41854	7258	7067	7258	7067	5519	0	5519
2	74451	37121	34885	30842	7223	7107	7223	7107	5519	0	5519
3	37360	18855	18230	16269	7254	7093	7254	7093	5519	0	5519
4	86597	42819	40791	30761	7260	7179	7260	7179	5519	0	5519
5	35705	17953	15794	14467	7022	6744	7022	6744	5519	0	5519
6	14563	7317	6969	5710	5694	5519	5694	5519	5519	0	5519
7	69894	34726	32081	26628	7256	7090	7256	7090	5519	0	5519
8	53794	26550	25502	22177	7258	7065	7258	7065	5519	0	5519
9	29945	15031	13907	11271	7186	6992	7186	6992	5519	0	5519
10	31349	15640	14494	12475	7241	6966	7241	6966	5519	0	5519
11	19603	9991	9471	7651	7239	7059	7239	7059	5519	0	5519
12	14353	7274	6809	5996	5954	5683	5954	5683	5519	0	5519
13	100103	50661	40413	34462	7226	6607	7226	6607	5519	0	5519
14	17310	8590	6979	5990	5968	5804	5968	5804	5519	0	5519
15	123354	62421	60666	54185	7254	7148	7254	7148	5519	0	5519
Minimum	14353	7274	6809	5710	5694	5519	5694	5519	5519	0	5519
Average	54237.9	27165.1	24942.3	21382.5	6952.9	6741.5	6952.9	6741.5	5519	0	5519
Maximum	123354	62421	60666	54185	7260	7179	7260	7179	5519	0	5519
Total	813568	407476	374135	320738	104293	101123	104293	101123	82785	0	82785

Table S3. Numbers of bacterial and fungal OTUs detected in all sampling areas.

Microbial group	Bacteria	Fungi
Khek specific	41	85
Than specific	197	121
San specific	182	243
Khek and Than	43	51
Khek and San	65	67
Than and San	240	122
All	263	147
Sum all taxa	1031	836

Figure S1. Map of Phitsanulok province, showing distribution of Podostemaceae. Colors indicate formations of type of rocks (Department of Mineral Resources. 2009) where the species were collected. Inset indicates Phitsanulok provinces in Thailand.

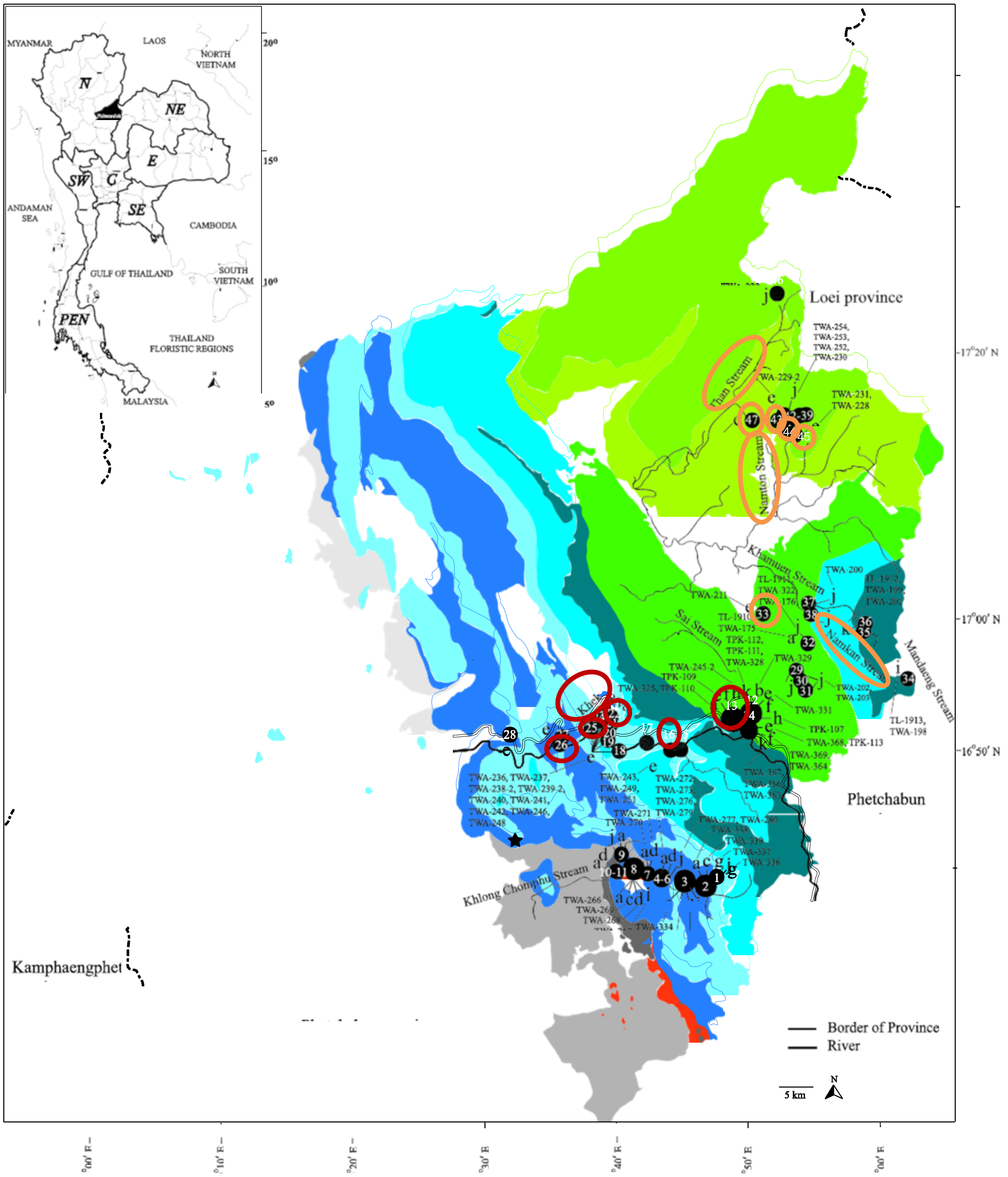

Letters (a–k) indicate species: a, *Dalzellia sparsa*; b, *Terniopsis heterostaminata*; c, *T. minor*; d, *Thawatchaia trilobata*; e, ***Hanseniella heterophylla***; f, *Hydrobryum bifoliatum*; g, *H. chompuense*; h, *H. kaengsophense*; i, *H. mandaengense*; j, *H. phetchabunense*; k, *H. Tardhuangense*. Solid circles indicate localities and numbers (1–46) within solid circles indicate waterfalls/rapids. Asterisk indicates Pai Si Thong waterfall where were not fond Podostemaceae. Red circles indicate sampling points (13, Kaeng Sopha Waterfall, 16, Kaeng Pakhao Krayang, 22, Kaeng Ratchamung, 25, Kaeng Yao, 26; Kaeng Tinthai Bon) in study area 1 (Khek river) and orange circles indicate sampling points (33, Kaeng Huataek, 43, Tadtinmee Waterfall; 44, Tadtam Waterfall; 45, Wanglum Waterfall, 47, Tintok Waterfall) in study area 2 (Than river: Than, Ton and Namkan rivers).

Source: Werukamkul P (2017). Taxonomic, molecular phylogenetic and ecological studies on Podostemaceae in the Phetchabun Mountain Range, Thailand (PhD thesis). Osaka City University, Japan.

Figure S2. Map of Loei province, showing distribution of Podostemaceae. Colors indicate formations of type of rocks (Department of Mineral Resources. 2008) where the species were collected. Inset indicates Loei provinces in Thailand.

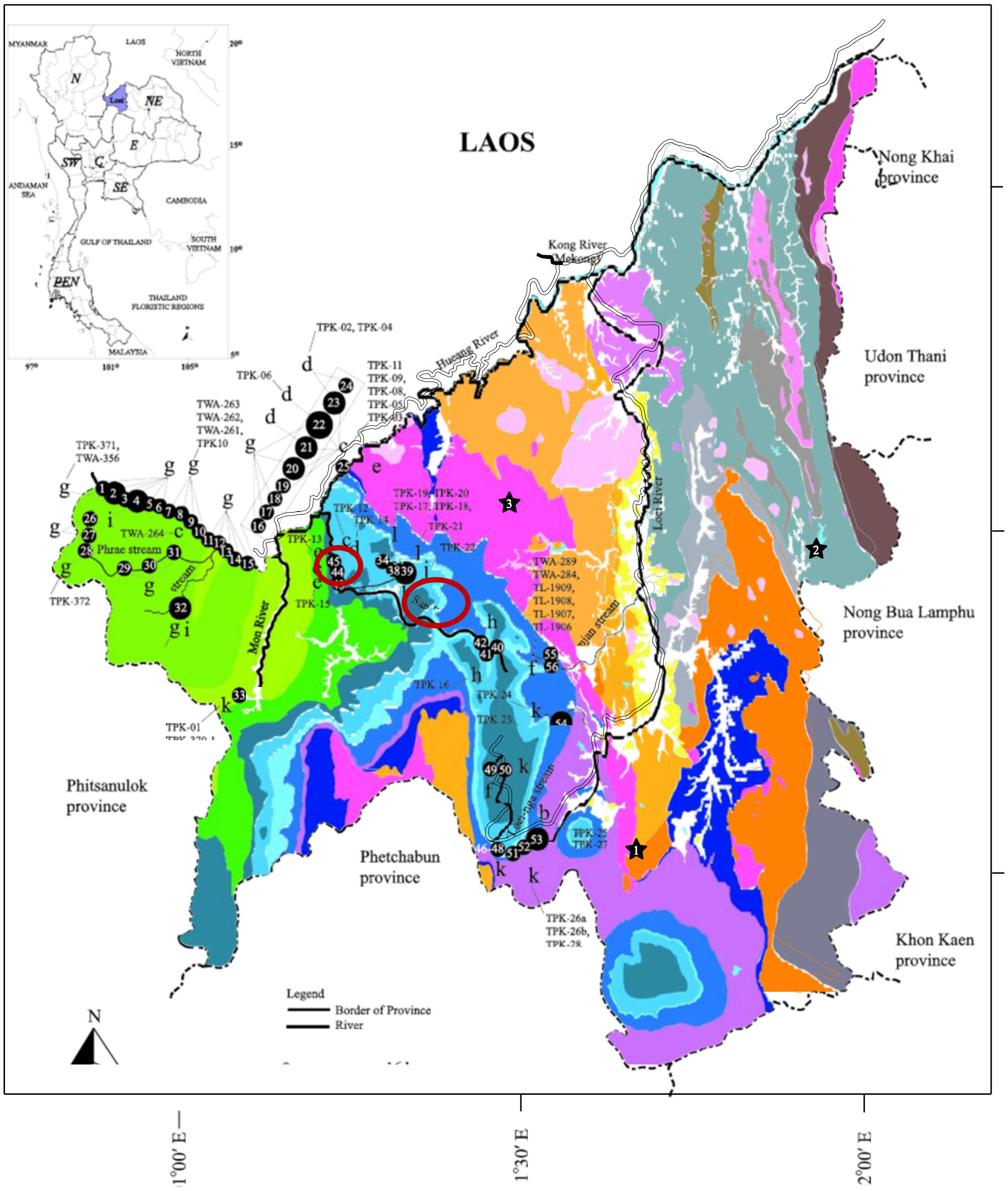

Letters (a–l) indicate species: a, *Dalzellia kailarsenii*; b, *Terniopsis filiformis*; c, *T. heterostaminata*; d, *Thawatchaia trilobata*; e, ***Hanseniella heterophylla***; f, *Hydrobryum japonicum*; g, *H. loeicum*; h, *H. phurueanum*; i, *H. tardhuangense*; j, *H. varium*; k, *H. vientianense*; l, *Polypleurum pluricostatum*. Solid circles indicate localities and numbers (1–56) within solid circles indicate waterfalls/rapids. Numbers (1–3) within stars indicate waterfalls where were not found Podostemaceae. 1, Suan Hom waterfall; 2, Tha Sawan waterfall; 3, Huai Khrai waterfall. Red circles indicate study area 3 (San river) and sampling points (43, Kaeng Gliang; 44, Kaeng Pak Nao).

Source: Werukamkul P (2017). Taxonomic, molecular phylogenetic and ecological studies on Podostemaceae in the Phetchabun Mountain Range, Thailand (PhD thesis). Osaka City University, Japan.

Figure S3. Individual rarefaction curves of bacteria (a) and fungi (b) detected in *Hanseniella heterophylla* samples.

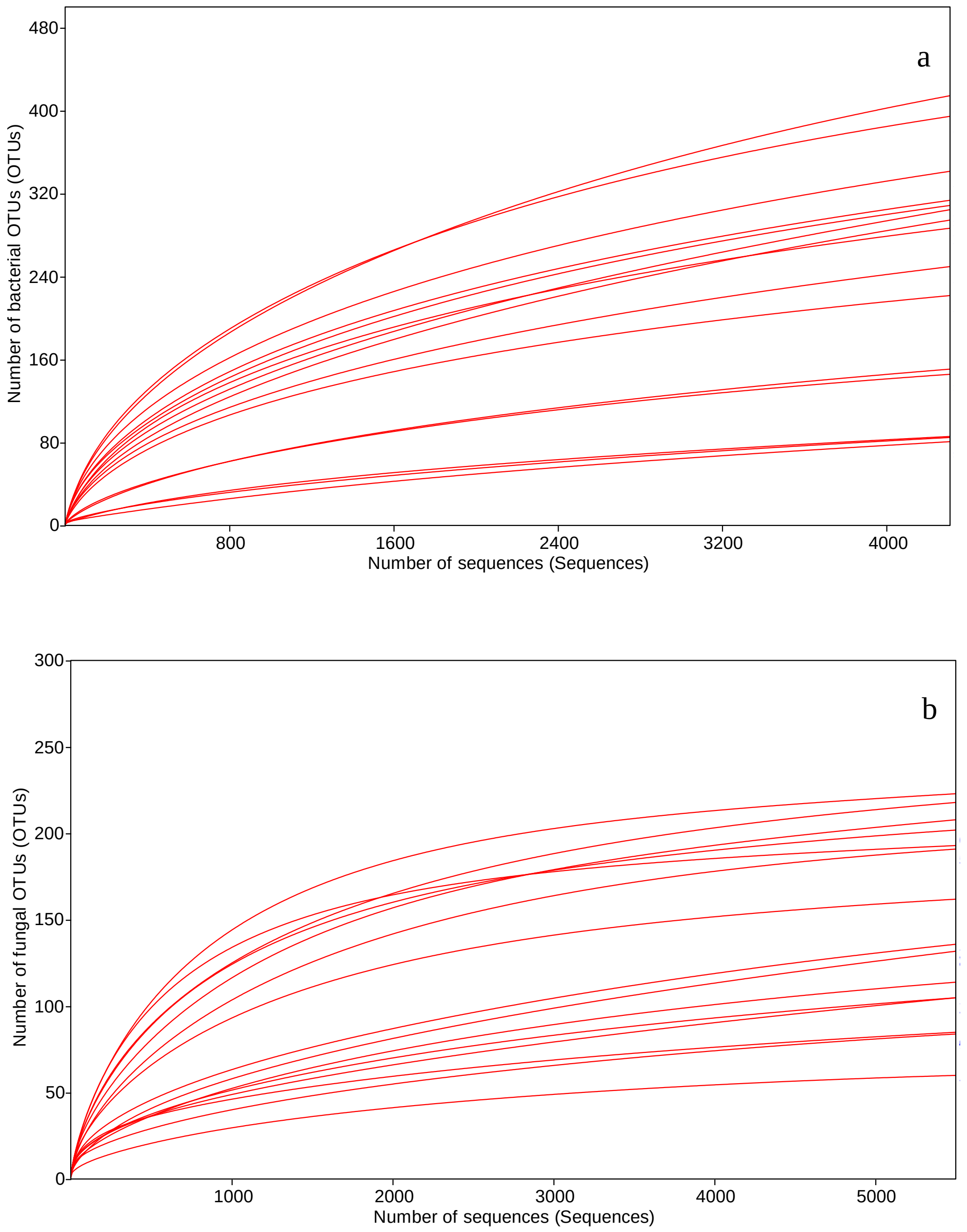

Figure S4. Effects of disturbance level and study areas on the diversity (Simpson index (1-dominance), calculated from rarified datasets from all bacterial and fungal OTUs (including rare taxa from original dataset)) (mean ± SE) of bacteria and fungi detected in *H. heterophylla*. Different letters above OTU richness bars within panels indicate significant differences (P < 0.05) according to one-way analysis of variance (ANOVA).

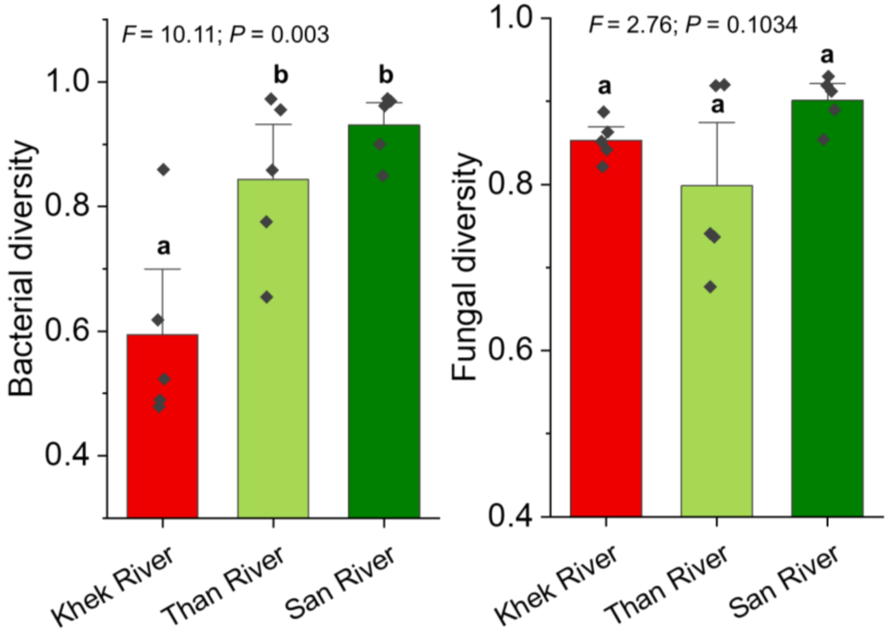

Figure S5. Effects of rock types on the OTU richness of bacteria in *H. heterophylla.* Different letters above OTU richness bars within a panel indicate significant differences *(P* < 0.05*)* according to one way ANOVA. Rock types: jpk: purple or purple-red siltstone with grey-green and yellow-brown sandstone, kkk: red and brown-red silt-and sandstones, ktpk: mixture of brown-red sandstone, siltstone, mudstone and conglomerate.

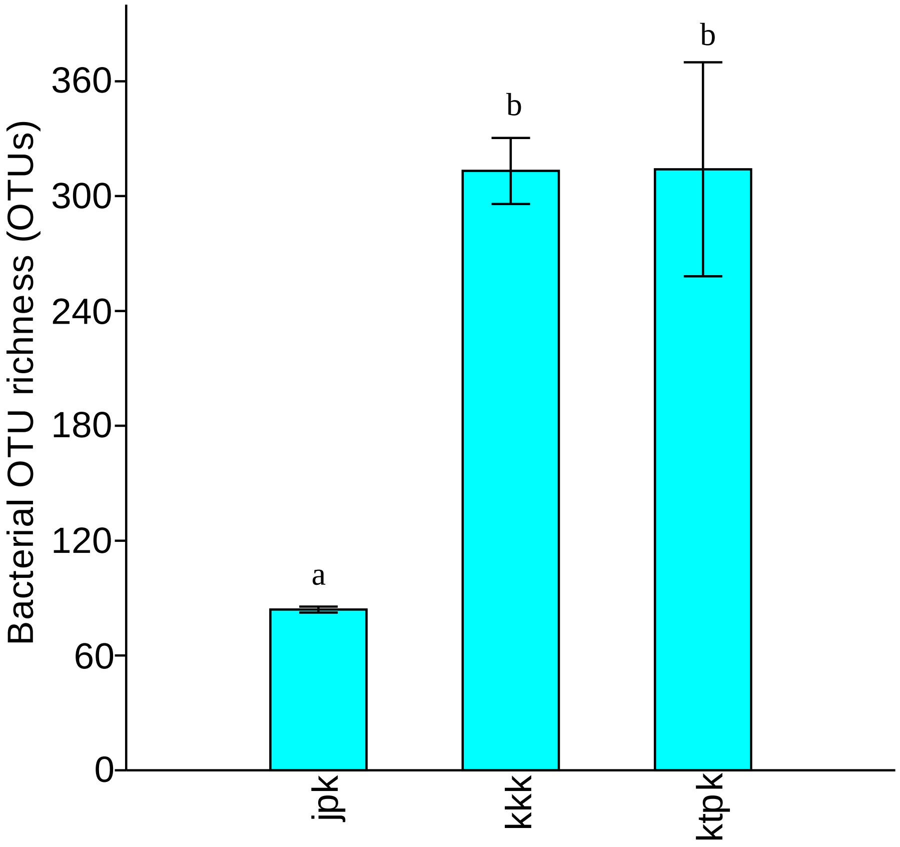

Figure S6. Heat map of all OTUs assigned as fungi with function detected in *H. heterophylla* plants (with minimum threshold of 40%, presence/absence data). K = Khek, T = Than and S = San.

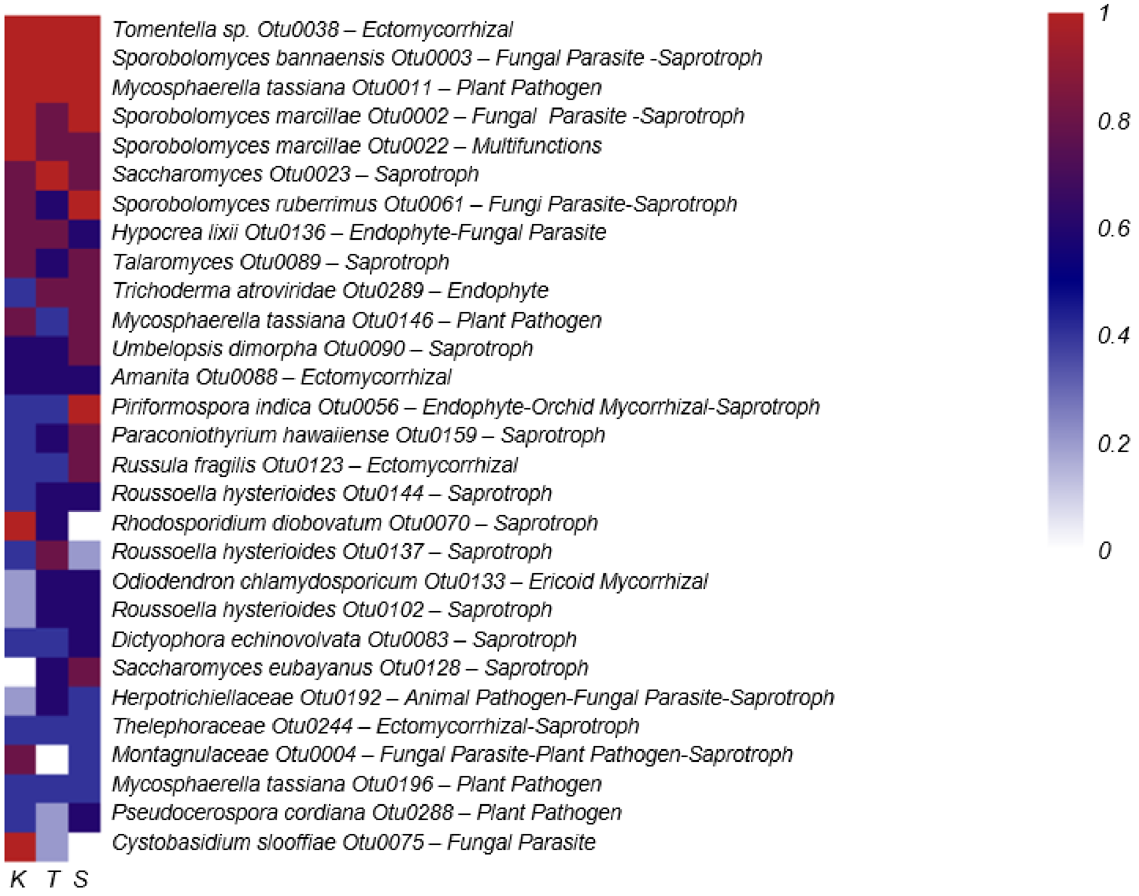

Supplement: Supplementary file 1 [file Data_Sheet_1.zip › Data Sheet 1/Supplementary Figures and Tables S1-S3.PDF]
